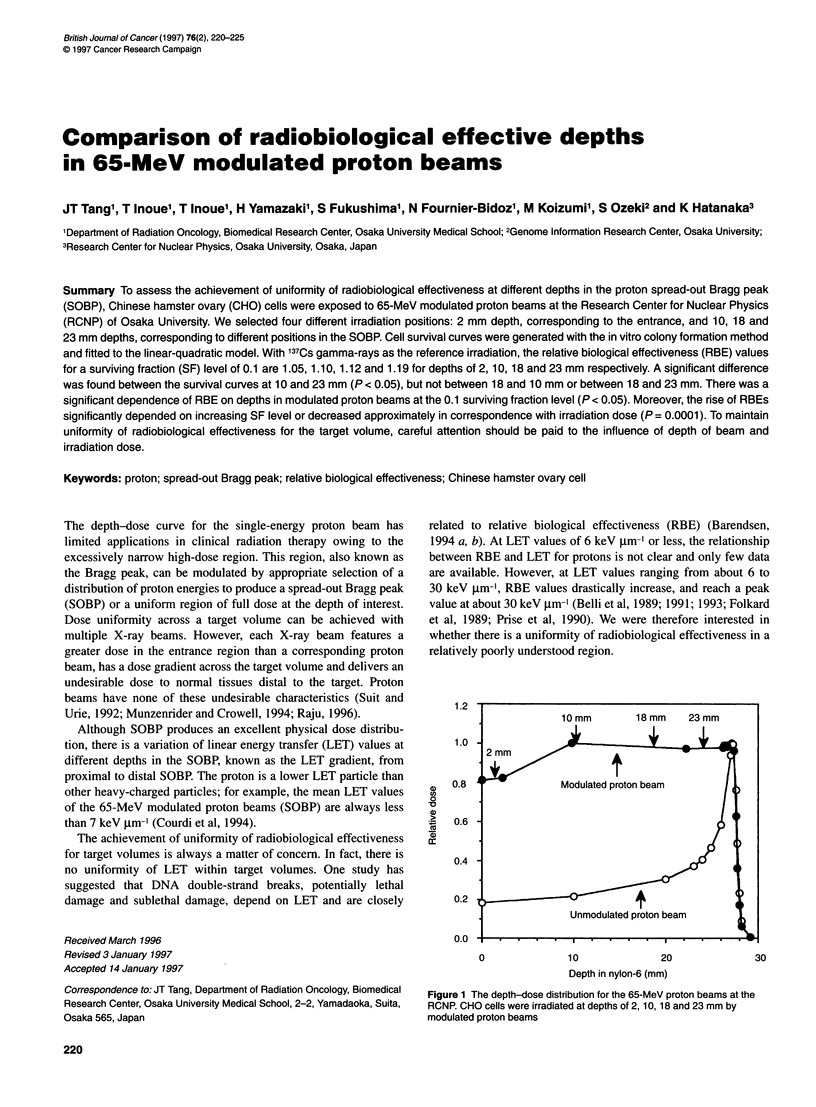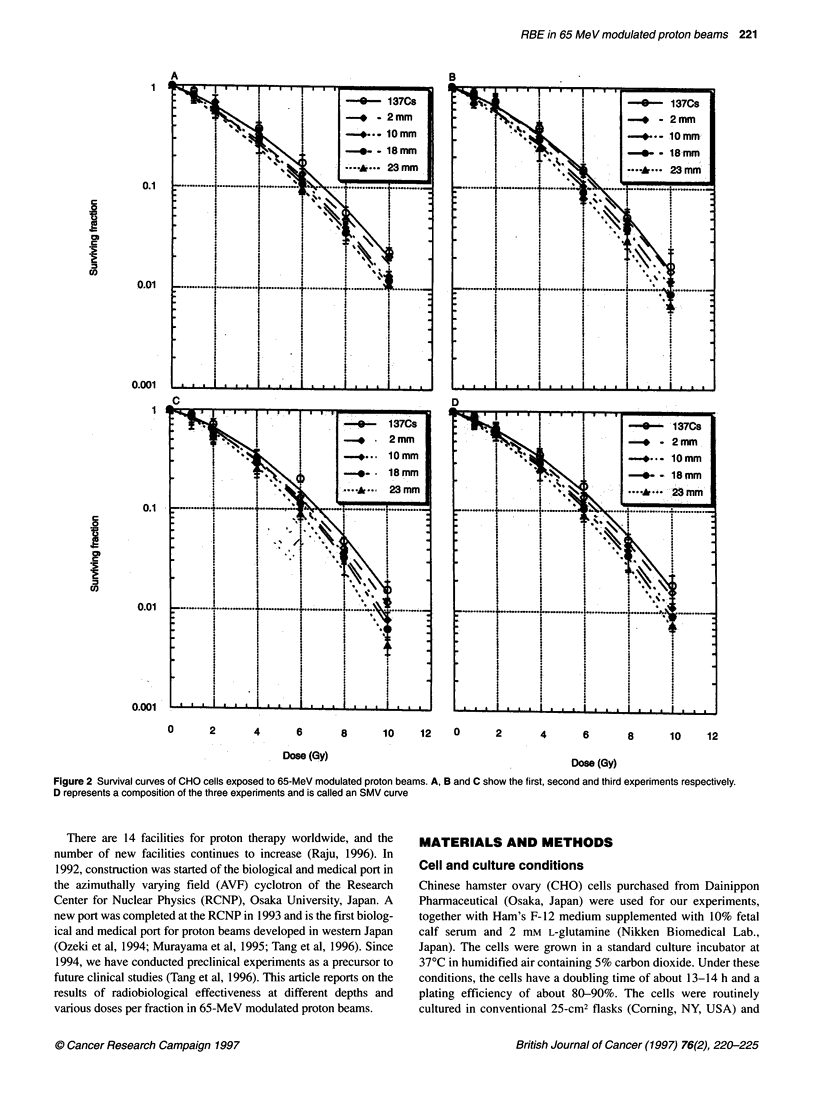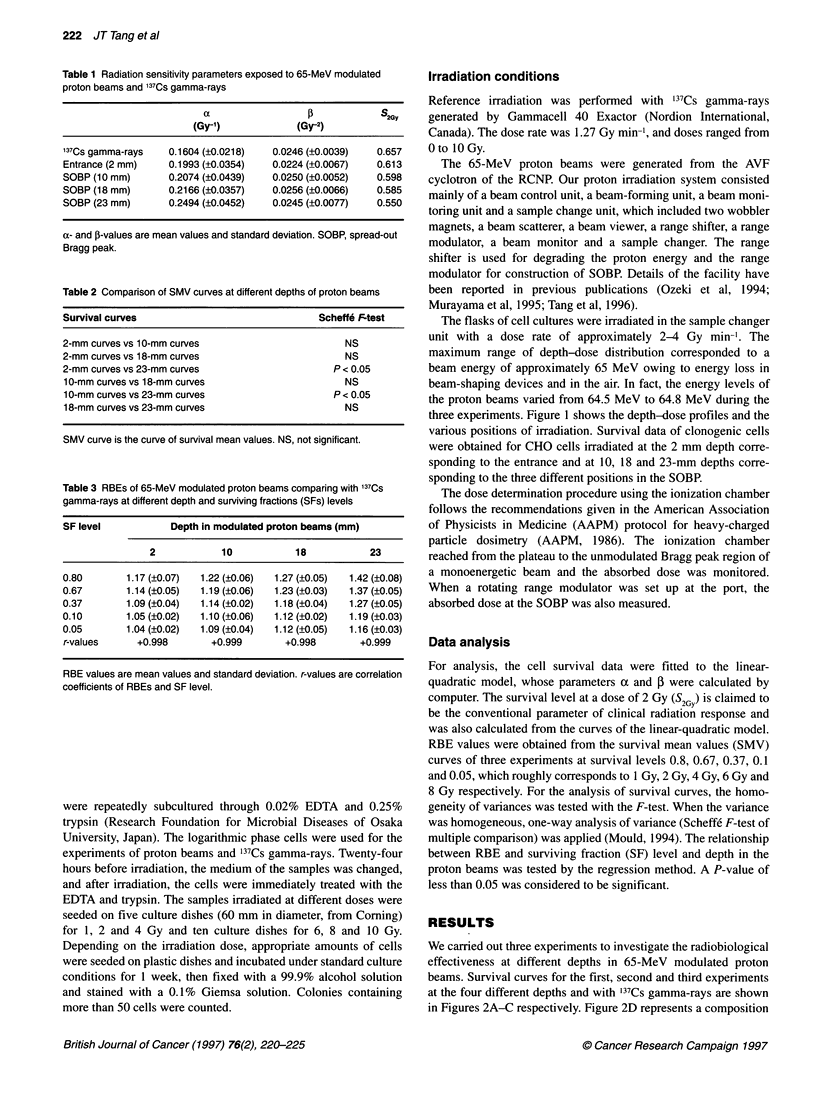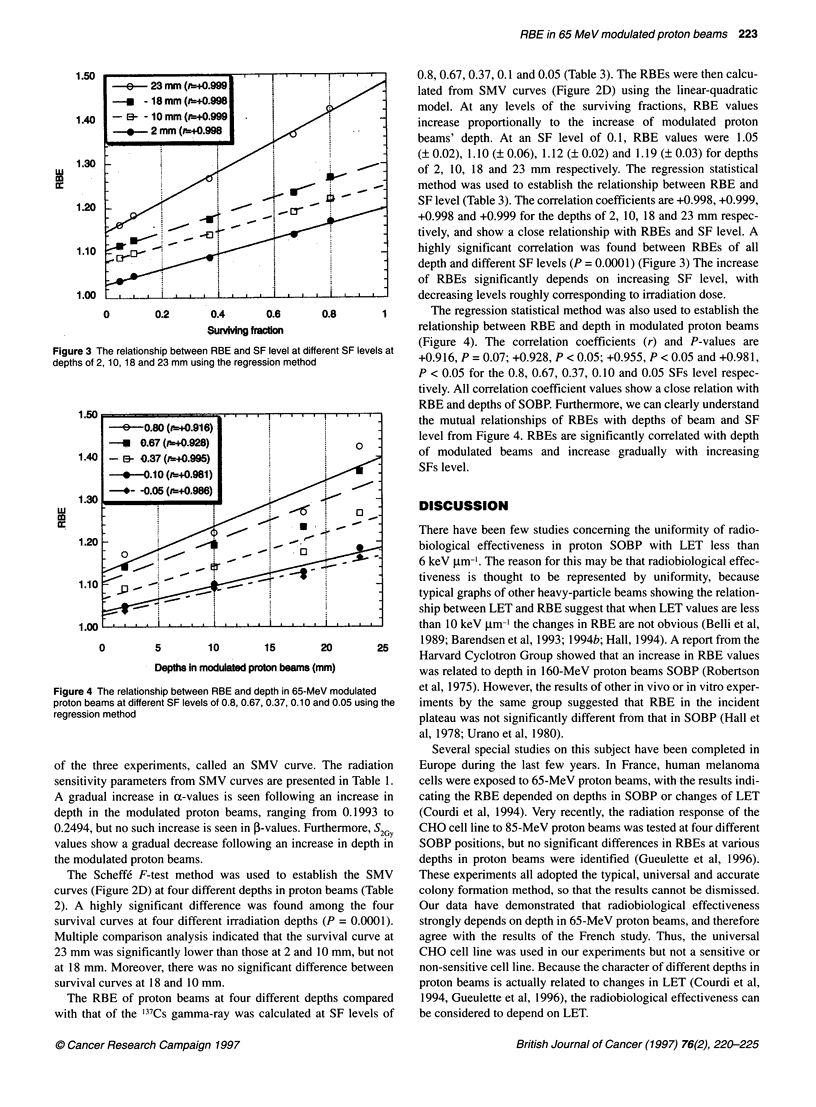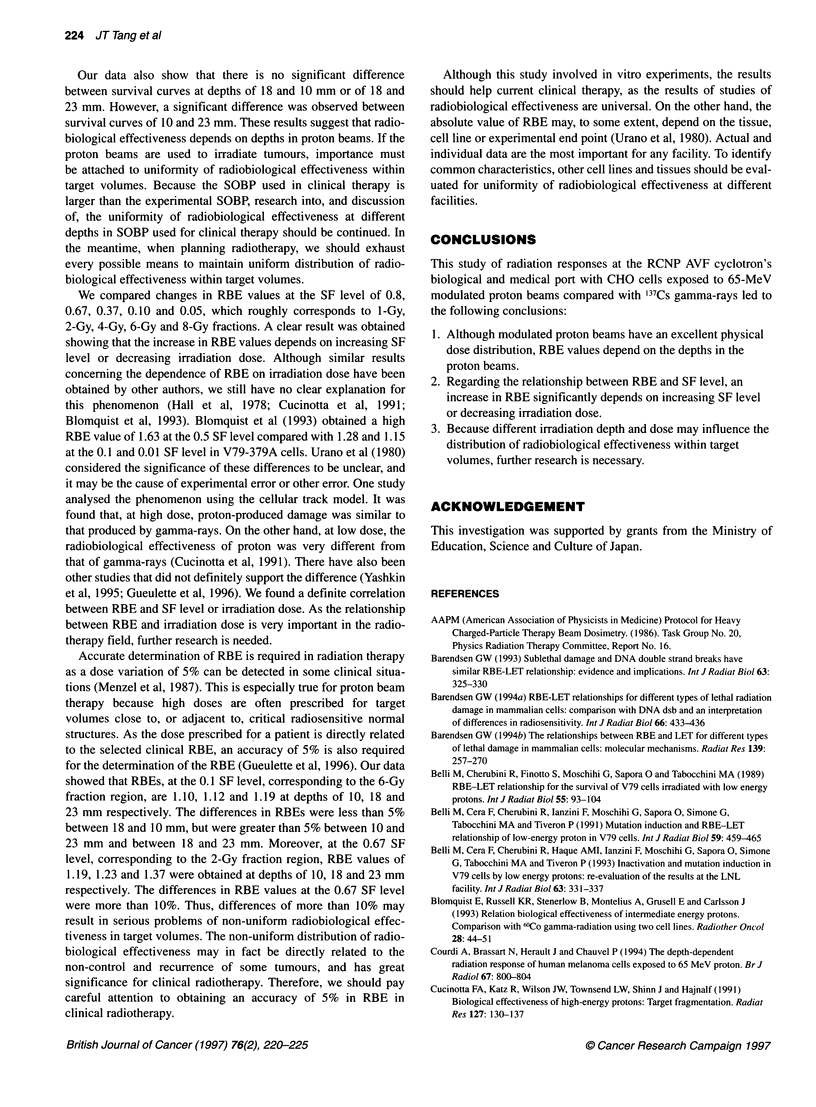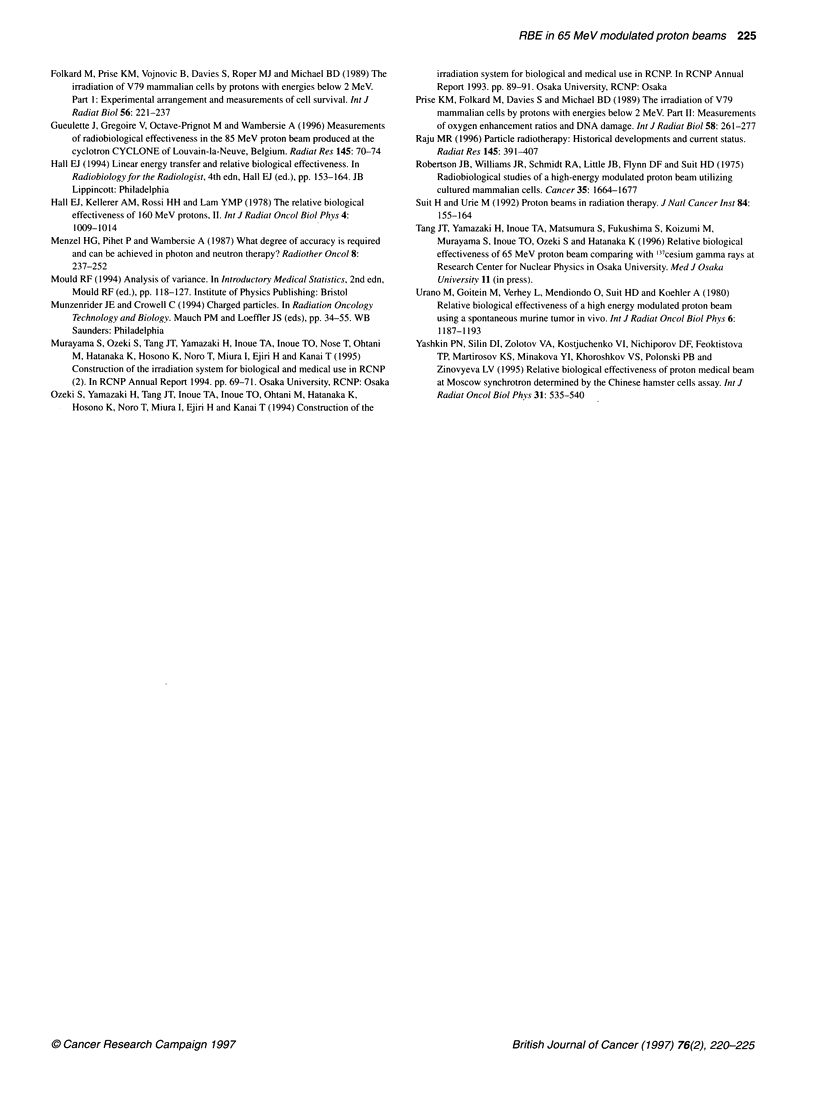# Comparison of radiobiological effective depths in 65-MeV modulated proton beams.

**DOI:** 10.1038/bjc.1997.365

**Published:** 1997

**Authors:** J. T. Tang, T. Inoue, T. Inoue, H. Yamazaki, S. Fukushima, N. Fournier-Bidoz, M. Koizumi, S. Ozeki, K. Hatanaka

**Affiliations:** Department of Radiation Oncology, Biomedical Research Center, Osaka University Medical School, Suita, Japan.

## Abstract

To assess the achievement of uniformity of radiobiological effectiveness at different depths in the proton spread-out Bragg peak (SOBP), Chinese hamster ovary (CHO) cells were exposed to 65-MeV modulated proton beams at the Research Center for Nuclear Physics (RCNP) of Osaka University. We selected four different irradiation positions: 2 mm depth, corresponding to the entrance, and 10, 18 and 23 mm depths, corresponding to different positions in the SOBP. Cell survival curves were generated with the in vitro colony formation method and fitted to the linear-quadratic model. With 137Cs gamma-rays as the reference irradiation, the relative biological effectiveness (RBE) values for a surviving fraction (SF) level of 0.1 are 1.05, 1.10, 1.12 and 1.19 for depths of 2, 10, 18 and 23 mm respectively. A significant difference was found between the survival curves at 10 and 23 mm (P < 0.05), but not between 18 and 10 mm or between 18 and 23 mm. There was a significant dependence of RBE on depths in modulated proton beams at the 0.1 surviving fraction level (P < 0.05). Moreover, the rise of RBEs significantly depended on increasing SF level or decreased approximately in correspondence with irradiation dose (P = 0.0001). To maintain uniformity of radiobiological effectiveness for the target volume, careful attention should be paid to the influence of depth of beam and irradiation dose.


British Journal of Cancer (1997) 76(2), 220–225
© 1997 Cancer Research Campaign

### Comparison of radiobiological effective depths in 65-MeV modulated proton beams

**JT Tang[1], T Inoue[1], T Inoue[1], H Yamazaki[1], S Fukushima[1], N Fournier-Bidoz[1], M Koizumi[1], S Ozeki[2] and K Hatanaka[3]**

[1]Department of Radiation Oncology, Biomedical Research Center, Osaka University Medical School; [2]Genome Information Research Center, Osaka University; [3]Research Center for Nuclear Physics, Osaka University, Osaka, Japan

**Summary** To assess the achievement of uniformity of radiobiological effectiveness at different depths in the proton spread-out Bragg peak (SOBP), Chinese hamster ovary (CHO) cells were exposed to 65-MeV modulated proton beams at the Research Center for Nuclear Physics (RCNP) of Osaka University. We selected four different irradiation positions: 2 mm depth, corresponding to the entrance, and 10, 18 and 23 mm depths, corresponding to different positions in the SOBP. Cell survival curves were generated with the in vitro colony formation method and fitted to the linear-quadratic model. With $^{137}Cs$ gamma-rays as the reference irradiation, the relative biological effectiveness (RBE) values for a surviving fraction (SF) level of 0.1 are 1.05, 1.10, 1.12 and 1.19 for depths of 2, 10, 18 and 23 mm respectively. A significant difference was found between the survival curves at 10 and 23 mm ($P < 0.05$), but not between 18 and 10 mm or between 18 and 23 mm. There was a significant dependence of RBE on depths in modulated proton beams at the 0.1 surviving fraction level ($P < 0.05$). Moreover, the rise of RBEs significantly depended on increasing SF level or decreased approximately in correspondence with irradiation dose ($P = 0.0001$). To maintain uniformity of radiobiological effectiveness for the target volume, careful attention should be paid to the influence of depth of beam and irradiation dose.

**Keywords:** proton; spread-out Bragg peak; relative biological effectiveness; Chinese hamster ovary cell

The depth–dose curve for the single-energy proton beam has limited applications in clinical radiation therapy owing to the excessively narrow high-dose region. This region, also known as the Bragg peak, can be modulated by appropriate selection of a distribution of proton energies to produce a spread-out Bragg peak (SOBP) or a uniform region of full dose at the depth of interest. Dose uniformity across a target volume can be achieved with multiple X-ray beams. However, each X-ray beam features a greater dose in the entrance region than a corresponding proton beam, has a dose gradient across the target volume and delivers an undesirable dose to normal tissues distal to the target. Proton beams have none of these undesirable characteristics (Suit and Urie, 1992; Munzenrider and Crowell, 1994; Raju, 1996).

Although SOBP produces an excellent physical dose distribution, there is a variation of linear energy transfer (LET) values at different depths in the SOBP, known as the LET gradient, from proximal to distal SOBP. The proton is a lower LET particle than other heavy-charged particles; for example, the mean LET values of the 65-MeV modulated proton beams (SOBP) are always less than 7 keV $\mu m^{-1}$ (Courdi et al, 1994).

The achievement of uniformity of radiobiological effectiveness for target volumes is always a matter of concern. In fact, there is no uniformity of LET within target volumes. One study has suggested that DNA double-strand breaks, potentially lethal damage and sublethal damage, depend on LET and are closely related to relative biological effectiveness (RBE) (Barendsen, 1994 *a, b*). At LET values of 6 keV $\mu m^{-1}$ or less, the relationship between RBE and LET for protons is not clear and only few data are available. However, at LET values ranging from about 6 to 30 keV $\mu m^{-1}$, RBE values drastically increase, and reach a peak value at about 30 keV $\mu m^{-1}$ (Belli et al, 1989; 1991; 1993; Folkard et al, 1989; Prise et al, 1990). We were therefore interested in whether there is a uniformity of radiobiological effectiveness in a relatively poorly understood region.

**Figure 1** The depth–dose distribution for the 65-MeV proton beams at the RCNP. CHO cells were irradiated at depths of 2, 10, 18 and 23 mm by modulated proton beams

*Received March 1996*
*Revised 3 January 1997*
*Accepted 14 January 1997*

*Correspondence to:* JT Tang, Department of Radiation Oncology, Biomedical Research Center, Osaka University Medical School, 2-2, Yamadaoka, Suita, Osaka 565, Japan

**Figure 2** Survival curves of CHO cells exposed to 65-MeV modulated proton beams. **A**, **B** and **C** show the first, second and third experiments respectively. **D** represents a composition of the three experiments and is called an SMV curve

There are 14 facilities for proton therapy worldwide, and the number of new facilities continues to increase (Raju, 1996). In 1992, construction was started of the biological and medical port in the azimuthally varying field (AVF) cyclotron of the Research Center for Nuclear Physics (RCNP), Osaka University, Japan. A new port was completed at the RCNP in 1993 and is the first biological and medical port for proton beams developed in western Japan (Ozeki et al, 1994; Murayama et al, 1995; Tang et al, 1996). Since 1994, we have conducted preclinical experiments as a precursor to future clinical studies (Tang et al, 1996). This article reports on the results of radiobiological effectiveness at different depths and various doses per fraction in 65-MeV modulated proton beams.

### MATERIALS AND METHODS

### Cell and culture conditions

Chinese hamster ovary (CHO) cells purchased from Dainippon Pharmaceutical (Osaka, Japan) were used for our experiments, together with Ham's F-12 medium supplemented with 10% fetal calf serum and 2 mM L-glutamine (Nikken Biomedical Lab., Japan). The cells were grown in a standard culture incubator at 37°C in humidified air containing 5% carbon dioxide. Under these conditions, the cells have a doubling time of about 13–14 h and a plating efficiency of about 80–90%. The cells were routinely cultured in conventional 25-cm² flasks (Corning, NY, USA) and

© Cancer Research Campaign 1997 

**Table 1** Radiation sensitivity parameters exposed to 65-MeV modulated proton beams and $^{137}Cs$ gamma-rays

| | α ($Gy^{-1}$) | β ($Gy^{-2}$) | $S_{2Gy}$ |
|---|---|---|---|
| $^{137}Cs$ gamma-rays | 0.1604 (±0.0218) | 0.0246 (±0.0039) | 0.657 |
| Entrance (2 mm) | 0.1993 (±0.0354) | 0.0224 (±0.0067) | 0.613 |
| SOBP (10 mm) | 0.2074 (±0.0439) | 0.0250 (±0.0052) | 0.598 |
| SOBP (18 mm) | 0.2166 (±0.0357) | 0.0256 (±0.0066) | 0.585 |
| SOBP (23 mm) | 0.2494 (±0.0452) | 0.0245 (±0.0077) | 0.550 |

α- and β-values are mean values and standard deviation. SOBP, spread-out Bragg peak.

**Table 2** Comparison of SMV curves at different depths of proton beams

| Survival curves | Scheffé *F*-test |
|---|---|
| 2-mm curves vs 10-mm curves | NS |
| 2-mm curves vs 18-mm curves | NS |
| 2-mm curves vs 23-mm curves | $P < 0.05$ |
| 10-mm curves vs 18-mm curves | NS |
| 10-mm curves vs 23-mm curves | $P < 0.05$ |
| 18-mm curves vs 23-mm curves | NS |

SMV curve is the curve of survival mean values. NS, not significant.

**Table 3** RBEs of 65-MeV modulated proton beams comparing with $^{137}Cs$ gamma-rays at different depth and surviving fractions (SFs) levels

| SF level | Depth in modulated proton beams (mm) | | | |
|---|---|---|---|---|
| | 2 | 10 | 18 | 23 |
| 0.80 | 1.17 (±0.07) | 1.22 (±0.06) | 1.27 (±0.05) | 1.42 (±0.08) |
| 0.67 | 1.14 (±0.05) | 1.19 (±0.06) | 1.23 (±0.03) | 1.37 (±0.05) |
| 0.37 | 1.09 (±0.04) | 1.14 (±0.02) | 1.18 (±0.04) | 1.27 (±0.05) |
| 0.10 | 1.05 (±0.02) | 1.10 (±0.06) | 1.12 (±0.02) | 1.19 (±0.03) |
| 0.05 | 1.04 (±0.02) | 1.09 (±0.04) | 1.12 (±0.05) | 1.16 (±0.03) |
| *r*-values | +0.998 | +0.999 | +0.998 | +0.999 |

RBE values are mean values and standard deviation. *r*-values are correlation coefficients of RBEs and SF level.

were repeatedly subcultured through 0.02% EDTA and 0.25% trypsin (Research Foundation for Microbial Diseases of Osaka University, Japan). The logarithmic phase cells were used for the experiments of proton beams and $^{137}Cs$ gamma-rays. Twenty-four hours before irradiation, the medium of the samples was changed, and after irradiation, the cells were immediately treated with the EDTA and trypsin. The samples irradiated at different doses were seeded on five culture dishes (60 mm in diameter, from Corning) for 1, 2 and 4 Gy and ten culture dishes for 6, 8 and 10 Gy. Depending on the irradiation dose, appropriate amounts of cells were seeded on plastic dishes and incubated under standard culture conditions for 1 week, then fixed with a 99.9% alcohol solution and stained with a 0.1% Giemsa solution. Colonies containing more than 50 cells were counted.

### Irradiation conditions

Reference irradiation was performed with $^{137}Cs$ gamma-rays generated by Gammacell 40 Exactor (Nordion International, Canada). The dose rate was 1.27 Gy $min^{-1}$, and doses ranged from 0 to 10 Gy.

The 65-MeV proton beams were generated from the AVF cyclotron of the RCNP. Our proton irradiation system consisted mainly of a beam control unit, a beam-forming unit, a beam monitoring unit and a sample change unit, which included two wobbler magnets, a beam scatterer, a beam viewer, a range shifter, a range modulator, a beam monitor and a sample changer. The range shifter is used for degrading the proton energy and the range modulator for construction of SOBP. Details of the facility have been reported in previous publications (Ozeki et al, 1994; Murayama et al, 1995; Tang et al, 1996).

The flasks of cell cultures were irradiated in the sample changer unit with a dose rate of approximately 2–4 Gy $min^{-1}$. The maximum range of depth–dose distribution corresponded to a beam energy of approximately 65 MeV owing to energy loss in beam-shaping devices and in the air. In fact, the energy levels of the proton beams varied from 64.5 MeV to 64.8 MeV during the three experiments. Figure 1 shows the depth–dose profiles and the various positions of irradiation. Survival data of clonogenic cells were obtained for CHO cells irradiated at the 2 mm depth corresponding to the entrance and at 10, 18 and 23-mm depths corresponding to the three different positions in the SOBP.

The dose determination procedure using the ionization chamber follows the recommendations given in the American Association of Physicists in Medicine (AAPM) protocol for heavy-charged particle dosimetry (AAPM, 1986). The ionization chamber reached from the plateau to the unmodulated Bragg peak region of a monoenergetic beam and the absorbed dose was monitored. When a rotating range modulator was set up at the port, the absorbed dose at the SOBP was also measured.

### Data analysis

For analysis, the cell survival data were fitted to the linear-quadratic model, whose parameters α and β were calculated by computer. The survival level at a dose of 2 Gy ($S_{2Gy}$) is claimed to be the conventional parameter of clinical radiation response and was also calculated from the curves of the linear-quadratic model. RBE values were obtained from the survival mean values (SMV) curves of three experiments at survival levels 0.8, 0.67, 0.37, 0.1 and 0.05, which roughly corresponds to 1 Gy, 2 Gy, 4 Gy, 6 Gy and 8 Gy respectively. For the analysis of survival curves, the homogeneity of variances was tested with the *F*-test. When the variance was homogeneous, one-way analysis of variance (Scheffé *F*-test of multiple comparison) was applied (Mould, 1994). The relationship between RBE and surviving fraction (SF) level and depth in the proton beams was tested by the regression method. A *P*-value of less than 0.05 was considered to be significant.

### RESULTS

We carried out three experiments to investigate the radiobiological effectiveness at different depths in 65-MeV modulated proton beams. Survival curves for the first, second and third experiments at the four different depths and with $^{137}Cs$ gamma-rays are shown in Figures 2A–C respectively. Figure 2D represents a composition

© Cancer Research Campaign 1997

**Figure 3** The relationship between RBE and SF level at different SF levels at depths of 2, 10, 18 and 23 mm using the regression method

**Figure 4** The relationship between RBE and depth in 65-MeV modulated proton beams at different SF levels of 0.8, 0.67, 0.37, 0.10 and 0.05 using the regression method of the three experiments, called an SMV curve. The radiation sensitivity parameters from SMV curves are presented in Table 1. A gradual increase in α-values is seen following an increase in depth in the modulated proton beams, ranging from 0.1993 to 0.2494, but no such increase is seen in β-values. Furthermore, $S_{2Gy}$ values show a gradual decrease following an increase in depth in the modulated proton beams.

The Scheffé *F*-test method was used to establish the SMV curves (Figure 2D) at four different depths in proton beams (Table 2). A highly significant difference was found among the four survival curves at four different irradiation depths ($P = 0.0001$). Multiple comparison analysis indicated that the survival curve at 23 mm was significantly lower than those at 2 and 10 mm, but not at 18 mm. Moreover, there was no significant difference between survival curves at 18 and 10 mm.

The RBE of proton beams at four different depths compared with that of the $^{137}Cs$ gamma-ray was calculated at SF levels of 0.8, 0.67, 0.37, 0.1 and 0.05 (Table 3). The RBEs were then calculated from SMV curves (Figure 2D) using the linear-quadratic model. At any levels of the surviving fractions, RBE values increase proportionally to the increase of modulated proton beams' depth. At an SF level of 0.1, RBE values were 1.05 (± 0.02), 1.10 (± 0.06), 1.12 (± 0.02) and 1.19 (± 0.03) for depths of 2, 10, 18 and 23 mm respectively. The regression statistical method was used to establish the relationship between RBE and SF level (Table 3). The correlation coefficients are +0.998, +0.999, +0.998 and +0.999 for the depths of 2, 10, 18 and 23 mm respectively, and show a close relationship with RBEs and SF level. A highly significant correlation was found between RBEs of all depth and different SF levels ($P = 0.0001$) (Figure 3) The increase of RBEs significantly depends on increasing SF level, with decreasing levels roughly corresponding to irradiation dose.

The regression statistical method was also used to establish the relationship between RBE and depth in modulated proton beams (Figure 4). The correlation coefficients ($r$) and $P$-values are +0.916, $P = 0.07$; +0.928, $P < 0.05$; +0.955, $P < 0.05$ and +0.981, $P < 0.05$ for the 0.8, 0.67, 0.37, 0.10 and 0.05 SFs level respectively. All correlation coefficient values show a close relation with RBE and depths of SOBP. Furthermore, we can clearly understand the mutual relationships of RBEs with depths of beam and SF level from Figure 4. RBEs are significantly correlated with depth of modulated beams and increase gradually with increasing SFs level.

### DISCUSSION

There have been few studies concerning the uniformity of radiobiological effectiveness in proton SOBP with LET less than 6 keV $\mu m^{-1}$. The reason for this may be that radiobiological effectiveness is thought to be represented by uniformity, because typical graphs of other heavy-particle beams showing the relationship between LET and RBE suggest that when LET values are less than 10 keV $\mu m^{-1}$ the changes in RBE are not obvious (Belli et al, 1989; Barendsen et al, 1993; 1994*b*; Hall, 1994). A report from the Harvard Cyclotron Group showed that an increase in RBE values was related to depth in 160-MeV proton beams SOBP (Robertson et al, 1975). However, the results of other in vivo or in vitro experiments by the same group suggested that RBE in the incident plateau was not significantly different from that in SOBP (Hall et al, 1978; Urano et al, 1980).

Several special studies on this subject have been completed in Europe during the last few years. In France, human melanoma cells were exposed to 65-MeV proton beams, with the results indicating the RBE depended on depths in SOBP or changes of LET (Courdi et al, 1994). Very recently, the radiation response of the CHO cell line to 85-MeV proton beams was tested at four different SOBP positions, but no significant differences in RBEs at various depths in proton beams were identified (Gueulette et al, 1996). These experiments all adopted the typical, universal and accurate colony formation method, so that the results cannot be dismissed. Our data have demonstrated that radiobiological effectiveness strongly depends on depth in 65-MeV proton beams, and therefore agree with the results of the French study. Thus, the universal CHO cell line was used in our experiments but not a sensitive or non-sensitive cell line. Because the character of different depths in proton beams is actually related to changes in LET (Courdi et al, 1994, Gueulette et al, 1996), the radiobiological effectiveness can be considered to depend on LET.

© Cancer Research Campaign 1997

Our data also show that there is no significant difference between survival curves at depths of 18 and 10 mm or of 18 and 23 mm. However, a significant difference was observed between survival curves of 10 and 23 mm. These results suggest that radiobiological effectiveness depends on depths in proton beams. If the proton beams are used to irradiate tumours, importance must be attached to uniformity of radiobiological effectiveness within target volumes. Because the SOBP used in clinical therapy is larger than the experimental SOBP, research into, and discussion of, the uniformity of radiobiological effectiveness at different depths in SOBP used for clinical therapy should be continued. In the meantime, when planning radiotherapy, we should exhaust every possible means to maintain uniform distribution of radiobiological effectiveness within target volumes.

We compared changes in RBE values at the SF level of 0.8, 0.67, 0.37, 0.10 and 0.05, which roughly corresponds to 1-Gy, 2-Gy, 4-Gy, 6-Gy and 8-Gy fractions. A clear result was obtained showing that the increase in RBE values depends on increasing SF level or decreasing irradiation dose. Although similar results concerning the dependence of RBE on irradiation dose have been obtained by other authors, we still have no clear explanation for this phenomenon (Hall et al, 1978; Cucinotta et al, 1991; Blomquist et al, 1993). Blomquist et al (1993) obtained a high RBE value of 1.63 at the 0.5 SF level compared with 1.28 and 1.15 at the 0.1 and 0.01 SF level in V79-379A cells. Urano et al (1980) considered the significance of these differences to be unclear, and it may be the cause of experimental error or other error. One study analysed the phenomenon using the cellular track model. It was found that, at high dose, proton-produced damage was similar to that produced by gamma-rays. On the other hand, at low dose, the radiobiological effectiveness of proton was very different from that of gamma-rays (Cucinotta et al, 1991). There have also been other studies that did not definitely support the difference (Yashkin et al, 1995; Gueulette et al, 1996). We found a definite correlation between RBE and SF level or irradiation dose. As the relationship between RBE and irradiation dose is very important in the radiotherapy field, further research is needed.

Accurate determination of RBE is required in radiation therapy as a dose variation of 5% can be detected in some clinical situations (Menzel et al, 1987). This is especially true for proton beam therapy because high doses are often prescribed for target volumes close to, or adjacent to, critical radiosensitive normal structures. As the dose prescribed for a patient is directly related to the selected clinical RBE, an accuracy of 5% is also required for the determination of the RBE (Gueulette et al, 1996). Our data showed that RBEs, at the 0.1 SF level, corresponding to the 6-Gy fraction region, are 1.10, 1.12 and 1.19 at depths of 10, 18 and 23 mm respectively. The differences in RBEs were less than 5% between 18 and 10 mm, but were greater than 5% between 10 and 23 mm and between 18 and 23 mm. Moreover, at the 0.67 SF level, corresponding to the 2-Gy fraction region, RBE values of 1.19, 1.23 and 1.37 were obtained at depths of 10, 18 and 23 mm respectively. The differences in RBE values at the 0.67 SF level were more than 10%. Thus, differences of more than 10% may result in serious problems of non-uniform radiobiological effectiveness in target volumes. The non-uniform distribution of radiobiological effectiveness may in fact be directly related to the non-control and recurrence of some tumours, and has great significance for clinical radiotherapy. Therefore, we should pay careful attention to obtaining an accuracy of 5% in RBE in clinical radiotherapy.

Although this study involved in vitro experiments, the results should help current clinical therapy, as the results of studies of radiobiological effectiveness are universal. On the other hand, the absolute value of RBE may, to some extent, depend on the tissue, cell line or experimental end point (Urano et al, 1980). Actual and individual data are the most important for any facility. To identify common characteristics, other cell lines and tissues should be evaluated for uniformity of radiobiological effectiveness at different facilities.

### CONCLUSIONS

This study of radiation responses at the RCNP AVF cyclotron's biological and medical port with CHO cells exposed to 65-MeV modulated proton beams compared with $^{137}Cs$ gamma-rays led to the following conclusions:

1. Although modulated proton beams have an excellent physical dose distribution, RBE values depend on the depths in the proton beams.
2. Regarding the relationship between RBE and SF level, an increase in RBE significantly depends on increasing SF level or decreasing irradiation dose.
3. Because different irradiation depth and dose may influence the distribution of radiobiological effectiveness within target volumes, further research is necessary.

### ACKNOWLEDGEMENT

This investigation was supported by grants from the Ministry of Education, Science and Culture of Japan.

### REFERENCES

AAPM (American Association of Physicists in Medicine) Protocol for Heavy Charged-Particle Therapy Beam Dosimetry. (1986). Task Group No. 20, Physics Radiation Therapy Committee, Report No. 16.

Barendsen GW (1993) Sublethal damage and DNA double strand breaks have similar RBE-LET relationship: evidence and implications. *Int J Radiat Biol* **63**: 325–330

Barendsen GW (1994*a*) RBE-LET relationships for different types of lethal radiation damage in mammalian cells: comparison with DNA dsb and an interpretation of differences in radiosensitivity. *Int J Radiat Biol* **66**: 433–436

Barendsen GW (1994*b*) The relationships between RBE and LET for different types of lethal damage in mammalian cells: molecular mechanisms. *Radiat Res* **139**: 257–270

Belli M, Cherubini R, Finotto S, Moschihi G, Sapora O and Tabocchini MA (1989) RBE–LET relationship for the survival of V79 cells irradiated with low energy protons. *Int J Radiat Biol* **55**: 93–104

Belli M, Cera F, Cherubini R, Ianzini F, Moschihi G, Sapora O, Simone G, Tabocchini MA and Tiveron P (1991) Mutation induction and RBE–LET relationship of low-energy proton in V79 cells. *Int J Radiat Biol* **59**: 459–465

Belli M, Cera F, Cherubini R, Haque AMI, Ianzini F, Moschihi G, Sapora O, Simone G, Tabocchini MA and Tiveron P (1993) Inactivation and mutation induction in V79 cells by low energy protons: re-evaluation of the results at the LNL facility. *Int J Radiat Biol* **63**: 331–337

Blomquist E, Russell KR, Stenerlow B, Montelius A, Grusell E and Carlsson J (1993) Relation biological effectiveness of intermediate energy protons. Comparison with $^{60}Co$ gamma-radiation using two cell lines. *Radiother Oncol* **28**: 44–51

Courdi A, Brassart N, Herault J and Chauvel P (1994) The depth-dependent radiation response of human melanoma cells exposed to 65 MeV proton. *Br J Radiol* **67**: 800–804

Cucinotta FA, Katz R, Wilson JW, Townsend LW, Shinn J and Hajnalf (1991) Biological effectiveness of high-energy protons: Target fragmentation. *Radiat Res* **127**: 130–137

© Cancer Research Campaign 1997

Folkard M, Prise KM, Vojnovic B, Davies S, Roper MJ and Michael BD (1989) The irradiation of V79 mammalian cells by protons with energies below 2 MeV. Part 1: Experimental arrangement and measurements of cell survival. *Int J Radiat Biol* **56**: 221–237

Gueulette J, Gregoire V, Octave-Prignot M and Wambersie A (1996) Measurements of radiobiological effectiveness in the 85 MeV proton beam produced at the cyclotron CYCLONE of Louvain-la-Neuve, Belgium. *Radiat Res* **145**: 70–74

Hall EJ (1994) Linear energy transfer and relative biological effectiveness. In *Radiobiology for the Radiologist*, 4th edn, Hall EJ (ed.), pp. 153–164. JB Lippincott: Philadelphia

Hall EJ, Kellerer AM, Rossi HH and Lam YMP (1978) The relative biological effectiveness of 160 MeV protons, II. *Int J Radiat Oncol Biol Phys* **4**: 1009–1014

Menzel HG, Pihet P and Wambersie A (1987) What degree of accuracy is required and can be achieved in photon and neutron therapy? *Radiother Oncol* **8**: 237–252

Mould RF (1994) Analysis of variance. In *Introductory Medical Statistics*, 2nd edn, Mould RF (ed.), pp. 118–127. Institute of Physics Publishing: Bristol

Munzenrider JE and Crowell C (1994) Charged particles. In *Radiation Oncology Technology and Biology*. Mauch PM and Loeffler JS (eds), pp. 34–55. WB Saunders: Philadelphia

Murayama S, Ozeki S, Tang JT, Yamazaki H, Inoue TA, Inoue TO, Nose T, Ohtani M, Hatanaka K, Hosono K, Noro T, Miura I, Ejiri H and Kanai T (1995) Construction of the irradiation system for biological and medical use in RCNP (2). In RCNP Annual Report 1994. pp. 69–71. Osaka University, RCNP: Osaka

Ozeki S, Yamazaki H, Tang JT, Inoue TA, Inoue TO, Ohtani M, Hatanaka K, Hosono K, Noro T, Miura I, Ejiri H and Kanai T (1994) Construction of the irradiation system for biological and medical use in RCNP. In RCNP Annual Report 1993. pp. 89–91. Osaka University, RCNP: Osaka

Prise KM, Folkard M, Davies S and Michael BD (1989) The irradiation of V79 mammalian cells by protons with energies below 2 MeV. Part II: Measurements of oxygen enhancement ratios and DNA damage. *Int J Radiat Biol* **58**: 261–277

Raju MR (1996) Particle radiotherapy: Historical developments and current status. *Radiat Res* **145**: 391–407

Robertson JB, Williams JR, Schmidt RA, Little JB, Flynn DF and Suit HD (1975) Radiobiological studies of a high-energy modulated proton beam utilizing cultured mammalian cells. *Cancer* **35**: 1664–1677

Suit H and Urie M (1992) Proton beams in radiation therapy. *J Natl Cancer Inst* **84**: 155–164

Tang JT, Yamazaki H, Inoue TA, Matsumura S, Fukushima S, Koizumi M, Murayama S, Inoue TO, Ozeki S and Hatanaka K (1996) Relative biological effectiveness of 65 MeV proton beam comparing with $^{137}$cesium gamma rays at Research Center for Nuclear Physics in Osaka University. *Med J Osaka University* **11** (in press).

Urano M, Goitein M, Verhey L, Mendiondo O, Suit HD and Koehler A (1980) Relative biological effectiveness of a high energy modulated proton beam using a spontaneous murine tumor in vivo. *Int J Radiat Oncol Biol Phys* **6**: 1187–1193

Yashkin PN, Silin DI, Zolotov VA, Kostjuchenko VI, Nichiporov DF, Feoktistova TP, Martirosov KS, Minakova YI, Khoroshkov VS, Polonski PB and Zinovyeva LV (1995) Relative biological effectiveness of proton medical beam at Moscow synchrotron determined by the Chinese hamster cells assay. *Int J Radiat Oncol Biol Phys* **31**: 535–540

© Cancer Research Campaign 1997